# Concomitant cemento-osseous dysplasia and aneurysmal bone cyst of the mandible: a rare case report with literature review

**DOI:** 10.1186/s12903-020-01264-7

**Published:** 2020-10-09

**Authors:** Han-Gyeol Yeom, Jung-Hoon Yoon

**Affiliations:** 1 Department of Oral and Maxillofacial Radiology, Daejeon Dental Hospital, Wonkwang University College of Dentistry, Daejeon, South Korea; 2 Department of Oral and Maxillofacial Pathology, Daejeon Dental Hospital, Wonkwang University College of Dentistry, Daejeon, South Korea

**Keywords:** Aneurysmal bone cyst, Fibro-osseous lesions, Cemento-osseous dysplasia

## Abstract

**Background:**

Concomitant cemento-osseous dysplasia (COD) and aneurysmal bone cyst (ABC) are rare in the head and neck region. In our search of the English language literature, we found only one case report describing the simultaneous occurrence of COD and ABC in the head and neck region. Here, we report a case of COD associated with ABC. Further, we performed a systematic search of the literature to identify studies on patients with COD associated with nonepithelial lined cysts of the jaws.

**Case presentation:**

The patient was a 32-year-old woman who was referred from a private dental clinic because of a cystic lesion below the mandibular right first molar. She had no pain or significant systemic disease. After performing panoramic radiography and cone-beam computed tomography, the imaging diagnosis was COD with a cystic lesion, such as ABC or solitary bone cyst. Excisional biopsy was performed, which revealed concomitant COD and ABC.

**Conclusion:**

This case of ABC associated with COD provides insight for the diagnostic process of radiographically mixed lesions with cystic changes.

## Background

Cemento-osseous dysplasia (COD) is a fibro-osseous lesion occurring in the tooth-bearing area of the jaws [1–3]. The etiology and pathogenesis are unknown, but it is thought to arise from the periodontal ligament because of the proximity of the lesions to the teeth and the formation of cementum-like calcifications [1, 3]. The lesions usually are asymptomatic, so COD is discovered in routine panoramic radiographs, and in most cases, no treatment is necessary [4]. Disease progression can be divided into three stages: osteolytic, mixed, and matured osteogenic [5]. Histopathologically, all forms of COD have a similar appearance; the lesion is not surrounded by a capsule, and the early osteolytic stage consists of fibrotic tissue rich in cells and vessels, with a little cementum-like deposits [3, 6]. As the maturation progresses over time, cementum-like calcifications and irregular trabeculae appear. In the final osteosclerotic stage, these structures connect to each other, and few cellular elements are visible [6].

The aneurysmal bone cyst (ABC) is a benign, osteolytic, rapidly growing, expansile lesion of the bone, which predominantly occurs in the metaphysis of long bones, such as the femur, tibia, and spine [7, 8]. Its occurrence in the jaws is uncommon [7]. Radiographically, ABCs are characterized by ballooned cortical expansion from unilocular to multilocular radiolucent lesions [8]. Histopathologically, ABCs are characterized by blood-filled spaces, separated by fibrous septa containing osteoclast-like giant cells [8]. The lesion develops either de novo as a true mesenchymal neoplasm, termed “primary ABC,” or secondary to a pre-existing bone lesion, termed “secondary ABC.” [9, 10] Secondary ABCs demonstrate similar pathologic characteristics as primary ABCs but have additional histologic findings indicating the presence of an additional coexisting lesion [10]. Approximately 70% of ABC cases are primary, whereas 30% are secondary [10, 11].

Nonepithelial lined cysts occasionally occur along with various bone lesions, mainly fibrous dysplasia, giant cell tumor, chondroblastoma, ossifying fibroma, benign osteoblastoma, COD, fibrous histiocytoma, fibrosarcoma [12]. These cysts include ABC, simple bone cyst (SBC), and non-specific cystic degeneration [12]. Concomitant COD and ABC lesions are rare in the head and neck region. In our search of the English-language literature, we found only one case report describing the occurrence of concomitant COD and ABC in the head and neck region [11]. Here, we report a case of COD associated with ABC. ABC associated with a benign fibro-osseous lesion has been investigated in previous studies [11], but no literature review has been conducted for COD associated with a nonepithelial cyst. Therefore, a systematic search of the literature was performed to identify studies reporting patients with COD associated with nonepithelial lined cysts of the jaws.

## Case presentation

The patient was a 32-year-old Korean woman referred because of a cystic lesion below the mandibular right first molar at a local dental clinic. She had no pain or significant systemic disease. Several clinical tests, including electric pulp test, were performed and the affected tooth showed the presence of pulp vitality. There was no tooth mobility.

**Fig. 1:**
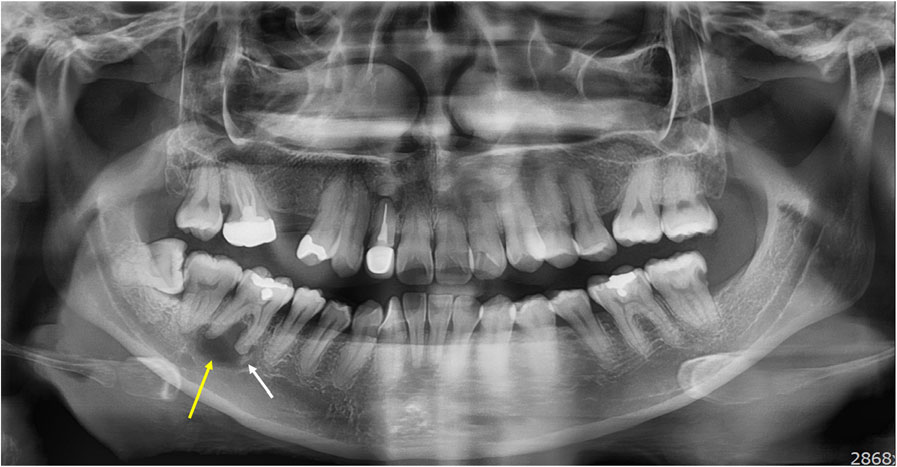
Panoramic radiograph showing a round mixed lesion below the mandibular right first molar (yellow arrow). The radiopaque portion was observed inside the radiolucent portion (white arrow)

Panoramic radiography revealed a round, mixed lesion below the mandibular right first molar (Fig. 1). The border of the lesion was clear, but no clear cortication was observed. The effects on the adjacent teeth and inferior alveolar canal were unclear. The lesion was generally radiolucent, but a mixed radiopaque portion was seen adjacent to the anterior boundary of the lesion, resembling the shape of a mixed lesion.

**Fig. 2:**
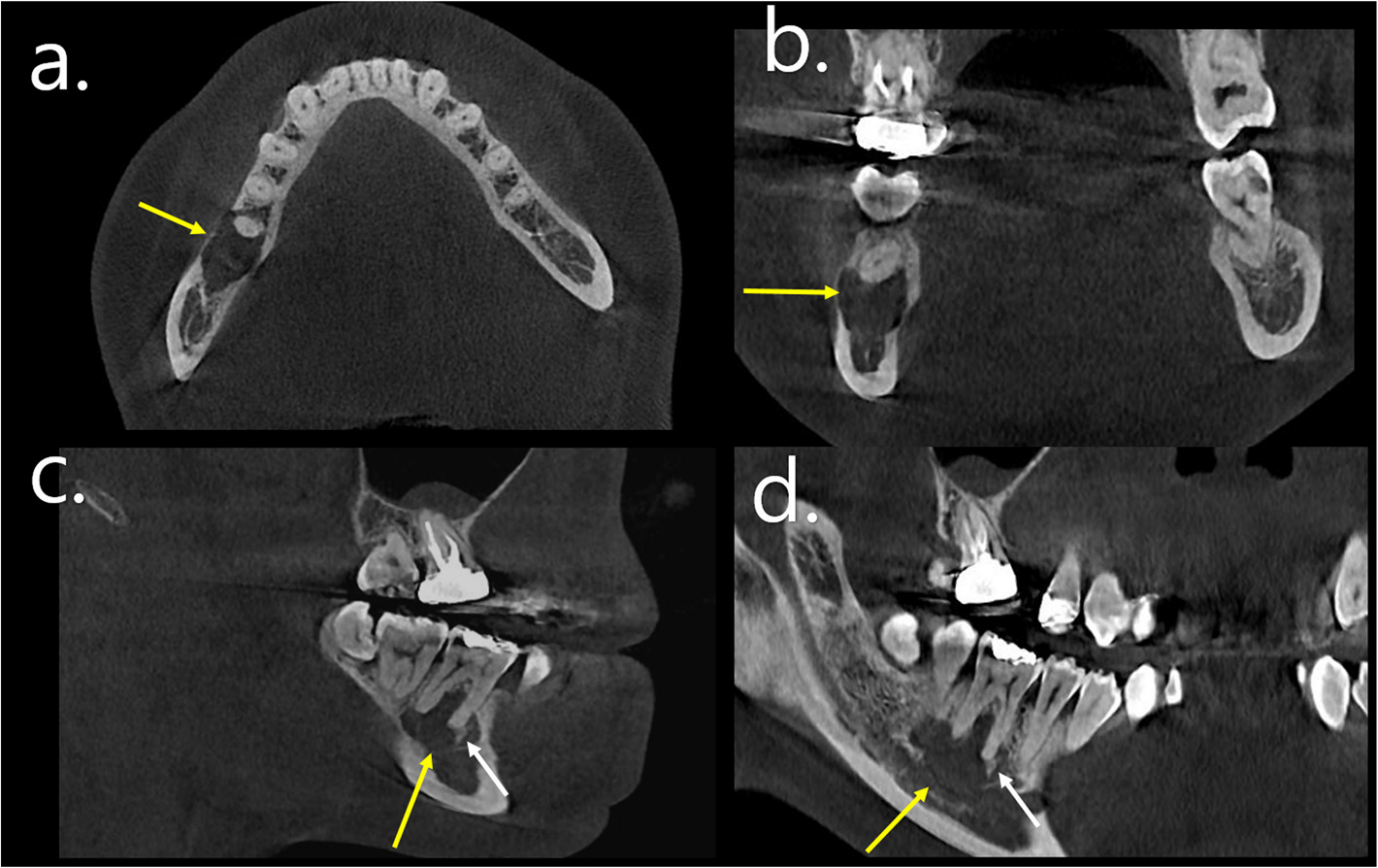
Cone-beam computed tomography scan of the patient. (a. axial, b. coronal, c. sagittal, d. panoramic view) A slightly ovoid lesion was observed anteriorly and backwards (yellow arrow). No apparent expansion pattern or deviation of the adjacent structures was observed. The radiopaque portion was observed inside the radiolucent portion (white arrow)

In the additionally obtained cone-beam computed tomography scan, a slightly ovoid lesion was observed anteriorly and backwards (Fig. 2). Likewise, the boundaries were clear, but no clear cortication was observed. Although thinning of the adjacent cortical bone was observed, no apparent expansion pattern was observed. The radiopaque portion inside the radiolucent portion was mixed at the anterior site of the lesion. The anterior part of the lesion showed the characteristics of COD. The middle and posterior parts of the lesion were suspected of cystic changes, showing low attenuation. The size of the area considered as a cystic portion was not sufficiently large, so it was difficult to distinguish it as a secondary cystic lesion. Considering all radiologic findings, the imaging diagnosis was COD with a cystic lesion, such as ABC or SBC.

**Fig. 3:**
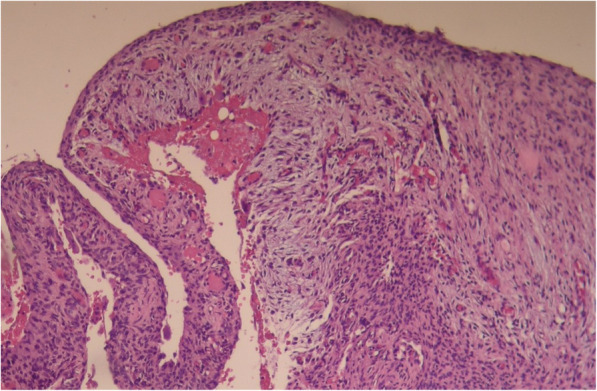
Cystic aneurysmal components show blood-filled cystic cavities lined with a thick membranous structure that comprises multinucleated giant cells. (H & E stain)

**Fig. 4:**
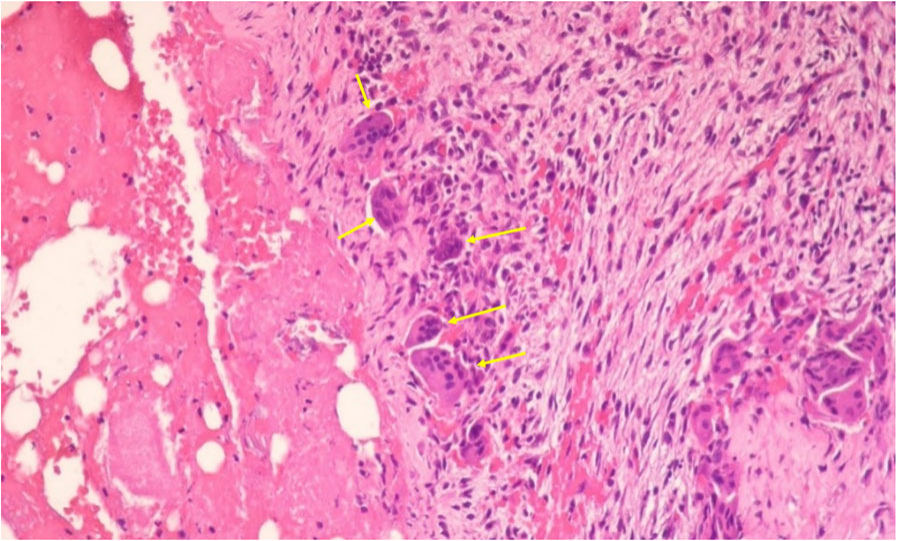
Cystic aneurysmal components composed of blood-filled spaces separated by connective tissue septa containing fibroblasts, osteoclast-type giant cells (yellow arrow).(H & E stain)

**Fig. 5:**
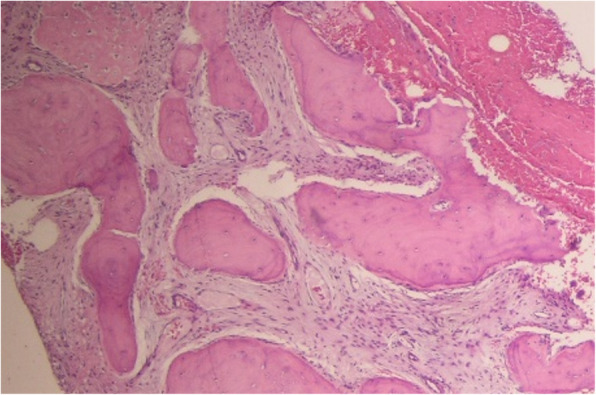
Anterior part of the lesion shows a sclerotic mass of cemento-osseous material. Stromal tissue is composed of spindle-shaped fibroblasts with small blood vessels (H & E stain)

**Fig. 6:**
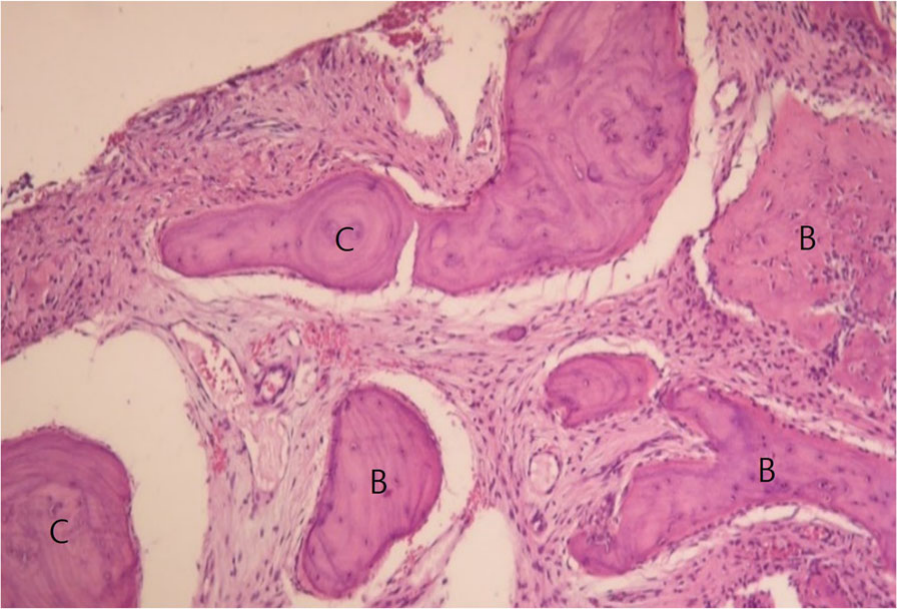
Cellular fibrous stroma contains an admixture of irregular trabeculae of bone and rounded globules of cementum-like material (C: cementicle, B: osseous portion). (H & E stain)

Surgical excision and histopathologic examination were performed. Histopathologically, stromal tissue was composed of spindle-shaped fibroblasts with small blood vessels (Fig. 3). Cystic aneurysmal components showed blood-filled cystic cavities lined with a thick membranous structure that comprised multinucleated giant cells and mononuclear cells (Fig. 4). The anterior part of the lesion showed a sclerotic mass of cemento-osseous material (Figs. 5, 6). Considering both histopathological and radiographic properties, the final diagnosis was concomitant COD and ABC.

One-year follow-up was done and there was no evidence of recurrence. Normal bone healing was seen without pathological findings.

## Discussion and conclusions

A systematic search of the literature was performed to identify studies reporting patients with COD associated with nonepithelial lined cysts of the jaws. English- and non-English-language papers were searched in PubMed/MEDLINE/Google Scholar databases and the gray literature using a combination of terms containing “cemento-osseous dysplasia” and containing “aneurysmal bone cyst” or “simple bone cyst” or “traumatic bone cyst” or “cyst” or “cystic lesion.” The search was carried out using the literature from 1985 to date. The focus of each reference varied, including a series of patients, case reports, and articles investigating the imaging appearance. Papers that reported epithelial lined cysts, such as a periapical cyst or dentigerous cyst, were excluded. We reviewed 116 papers and finally selected 14. Table 1 summarizes the data of the available papers.

**Table 1:** Table 1 Summary of literature review: patients with COD associated with nonepithelial lined cysts of the jaws

Authors	Year	Concomitant	Sample size	Sex	Age (years)	Site	Pain	Treatment
Higuchi et al. [13]	1988	COD & SBC	4	F	40	Left third molar	N	Excision
				F	31	Left second molar	N	Excision
				F	42	Left second premolar to left second molar	N	Excision
				F	49	Left canine	N	Excision
Miyauchi et al. [14]	1995	COD & SBC	1	F	40	Right premolar-retromolar/right canine to left first premolar	Y	Excision
Wakasa et al. [15]	2002	COD & SBC	1	F	34	Right retromolar/right premolar-molar/left premolar-central incisor/left molar	Y	Curettage
Mupparapu et al. [16]	2005	COD & SBC	1	F	41	Right premolar-molar	N	Curettage
Mahomed et al. [17]	2005	COD & SBC	7	F	43	Right molar	Y	Excision
				F	54	Left ramus-right ramus	Y	Excision
				F	48	Left molar to right molar	N	Excision
				F	26	Right molar	N	Excision
				F	35	Left third molar	Y	Excision
				F	48	Both molars	Y	Excision
				F	42	Right molar	Y	Excision
Mupparapu et al. [18]	2008	COD & SBC	1	F	39	Right mandibular/left mandibular 2	Y	Curettage
Martini et al. [19]	2010	COD & SBC	3	F	66	Left mandibular molar	N	–
				F	11	Left premolar-molar	N	–
				F	42	Left premolar-molar	N	–
Rao et al. [20]	2011	COD & SBC	1	F	41	Right molar	Y	Excision
Chadwick et al. [21]	2011	COD & SBC	23	F-20	47	–	–	–
				M-3	41.5	–	–	–
Eroglu et al. [22]	2016	COD & SBC	1	F	23	Left premolar	N	Decompression
Jacomacci et al. [11]	2017	COD & ABC	1	F	41	Right premolar	N	Excision
Singer et al. [23]	2019	COD & SBC	1	F	59	Anterior mandible	Y	Excision
Chinckr et al. [24]	2020	COD & SBC	1	F	40	Right mandibular body	Y	Saucerization

We found 46 patients with a female predilection (43 women vs 3 men). Forty-five patients had concomitant COD and SBC, while only one patient had concomitant COD and ABC. The mean age was 43.47 years, ranging from 11 to 66 years. The mandibular posterior area (premolar, molar, retromolar, and ramus areas) was the most frequently reported site (21 cases: mandibular posterior area; 2 cases: mandibular canine; 23 cases: not mentioned). Clinically, 11 patients presented with pain, while 12 patients presented with no pain. The pain status of 23 patients was not mentioned.

Both COD and ABC are non-neoplastic intraosseous lesions. The concomitant occurrence of these lesions could be explained by the probable pathogenesis of ABC. The stroma of COD often displayed characteristic cavernous-like vascularity that was almost always associated with bony trabeculae [6]. Free hemorrhage was frequently interspersed in the artifactual spaces throughout COD [6]. ABC formation could be attributed to a local circulatory disturbance causing a markedly elevated venous pressure and the development of enlarged vascular spaces within the affected bone [7, 25]. This hemodynamic disturbance could be secondary to local trauma or the presence of a pre-existing lesion in the affected bone [7, 25]. Clinically, COD usually presents slow-growing, painless lesions, whereas the ABC presents a rapidly growing, painful lesion. Our patient had no pain, but the radiograph revealed a lesion.

Our literature search demonstrated only one case report of concomitant COD and ABC [11], so an accurate analysis is difficult with only two cases. Both patients were women, and the ages were 32 and 41 years, respectively. In case of concomitant COD with a cystic lesion, SBC was reported as a cystic lesion more than ABC. The SBC is a pseudocyst similar to ABC in various aspects: most frequently found in the long bones (50%) and spine (20%) but rarely in the jaw bones (2%). However, the entity of ABC tends to have a more aggressive clinical behavior compared to SBC.

Compared to one previously reported concomitant COD and ABC case, our patient showed the anterior part of the mixed lesion and the posterior cystic portion. Buccolingual expansion was unclear in the cystic portion. Since the cystic portion was small, it was difficult to distinguish ABC or SBC in the radiologic findings. Pathologically, the COD portion was observed on one side, and most of the lesions showed the characteristics of ABC. The part where the COD was clearly diagnosable was considered to be the anterior part of the lesion observed in the radiologic mixed lesion.

The imaging findings of the previous case showed the characteristics of ABC with clear buccolingual expansion. In the pathologic findings presented, the characteristics of COD were generally observed, and in some of them, the ABC findings were visible. Both lesions were diagnosed as concomitant COD and ABC, but the imaging and pathologic findings showed some differences among similar tendencies. Although there are various limitations to generalization due to the small number of cases, so, but we are expecting to better understand and diagnose nonepithelial cystic lesions associated with COD by reporting this case, in which an ABC and COD were present in association.

## Data Availability

All data analyzed during this study are included in this published article.

## Abbreviations

COD: Cemento-osseous dysplasia
ABC: Aneurysmal bone cyst
SBC: Simple bone cyst
